# Spontaneous Rupture of the Membranes at Full Term

**Published:** 1885-06

**Authors:** 


SPONTANEOUS RUPTURE OF THE MEMBRANES AT FULL TERM.

The results of his observations in fifty cases of spontaneous rupture of the membranes are given in "The Association of the Medical Sciences for Apr l," by Dr. G. W. H. Kemper.

The cases occurred in seven hundred cases of pregnancy. His conclusions are: That spontaneous rupture of the membranes at full term, before the advent of labor pains, is quite common about once in every fourteen pregnancies. Labor usually comes on at once or soon after, although there may be delay for several days or even weeks. The causes are not known, but the inherent tendency of some women to this accident is shown by its repeated occurrence in their pregnancies. The effects upon the duration or severity of labor, or upon the mortality of either mother or child, are nil. The duration of labor is probably shorter in cases where some time elapses before pains begin. The plan of treatment is to place the patient in the recumbent posture and wait. All mechanical or other means used to excite labor pains are hurtful and meddlesome.

Dr. Kemper does not believe that the old cry of "dry labor" is justified in these cases, or that any ills in childbed are to be expected in consequence of this casualty.

